# Differential Progression of Regional Hippocampal Atrophy in Aging and Parkinson’s Disease

**DOI:** 10.3389/fnagi.2018.00325

**Published:** 2018-10-11

**Authors:** Carme Uribe, Barbara Segura, Hugo C. Baggio, Anna Campabadal, Alexandra Abos, Yaroslau Compta, Maria Jose Marti, Francesc Valldeoriola, Nuria Bargallo, Carme Junque

**Affiliations:** ^1^Medical Psychology Unit, Department of Medicine, Institute of Neuroscience, University of Barcelona, Barcelona, Spain; ^2^Centro de Investigación Biomédica en Red Sobre Enfermedades Neurodegenerativas (CIBERNED), Hospital Clínic de Barcelona, Barcelona, Spain; ^3^Institute of Biomedical Research August Pi i Sunyer (IDIBAPS), Barcelona, Spain; ^4^Parkinson’s Disease and Movement Disorders Unit, Neurology Service, Hospital Clínic de Barcelona, Institute of Neuroscience, University of Barcelona, Barcelona, Spain; ^5^Centre de Diagnòstic per la Imatge, Hospital Clínic, Barcelona, Spain

**Keywords:** hippocampal subfields, MRI, Parkinson’s disease, longitudinal assessment, memory impairment, aging

## Abstract

Hippocampal subfields have different vulnerability to the degenerative processes related to aging, amnestic mild cognitive impairment (MCI) and Alzheimer’s disease (AD), but the temporal evolution in Parkinson’s disease (PD) is unknown. The purposes of the current work are to describe regional hippocampal changes over time in a sample of PD patients classified according to their baseline cognitive status and to relate these changes to verbal memory loss. T1-weighted images and verbal memory assessment were obtained at two separate time points (3.8 ± 0.4 years apart) from 28 PD with normal cognition (PD-NC), 16 PD with MCI (PD-MCI) and 21 healthy controls (HCs). FreeSurfer 6.0 automated pipeline was used to segment the hippocampus into 12 bilateral subregions. Memory functions were measured with Rey’s Auditory Verbal learning test (RAVLT). We found significant reductions in cornu ammonis 1 (CA1) over time in controls as well as in PD subgroups. Right whole-hippocampal volumes showed time effects in both PD groups but not in controls. PD-NC patients also displayed time effects in the left hippocampal tail and right parasubiculum. Regression analyses showed that specific hippocampal subfield volumes at time 1 predicted almost 60% of the variability in RAVLT delayed-recall score decline. Changes in several hippocampal subregions also showed predictive value for memory loss. In conclusion, CA1 changes in PD were similar to those that occur in normal aging, but PD patients also had more decline in both anterior and posterior hippocampal segments with a more pronounced atrophy of the right hemisphere. Hippocampal segments are better predictors of changes in memory performance than whole-hippocampal volumes.

## Introduction

Hippocampal atrophy is a key finding in neurodegenerative diseases (Camicioli et al., 2003; Small et al., 2011; Bartsch and Wulff, 2015; Yang and Yu, 2017), although it is also present in healthy aging (Fjell et al., 2014). In neuroimaging studies, the hippocampus has traditionally been assessed as a single component, but more advanced techniques have allowed studying the hippocampus as a complex structure with specific regional vulnerability to aging and subtypes of dementia (Small et al., 2011).

Extensive previous literature consistently reports region 1 of the cornu ammonis (area CA1), the subiculum (Mueller et al., 2010) and area CA3 (Pereira et al., 2014; Wisse et al., 2014) as the regions that are most vulnerable to degeneration in normal aging and Alzheimer’s disease (AD; de Flores et al., 2015). In Parkinson’s disease (PD), hippocampal atrophy has been associated with dementia (Junqué et al., 2005; Summerfield et al., 2005; Ibarretxe-Bilbao et al., 2008), although volume reductions can also be detectable in non-demented PD (Junqué et al., 2005; Pereira et al., 2013) and even in unmedicated patients (Noh et al., 2014).

The detection of regional hippocampal atrophy and its association with memory decline is of high interest in PD since memory impairment has been described as a risk factor for dementia (Levy et al., 2002). Total hippocampal volumes correlated with learning tasks (Pereira et al., 2013); recognition memory, on the other hand, has been associated with left hippocampal atrophy (Camicioli et al., 2003). More recently, volume reductions in some subregions such as areas CA2–3 and CA4 and the dentate gyrus (DG) have been linked to verbal learning impairment in PD (Engvig et al., 2012; Pereira et al., 2013). Moreover, CA2–3 atrophy has been found to discriminate healthy controls (HCs) from amnestic mild cognitive impairment (MCI) patients better than global hippocampal volumes (Hanseeuw et al., 2011).

In the last 3 years, thanks to the development of automated segmentations tools, it has become possible to divide the hippocampus into 12 bilateral segments based on a statistical atlas built upon ultra-high resolution *ex-vivo* MRI data (Iglesias et al., 2015). To our knowledge, only one published study investigated differences in percentage change over a 1.5-year follow-up between PD patients with normal cognition (PD-NC) and with MCI (PD-MCI) in hippocampal subfields also using this automated segmentation pipeline (Foo et al., 2016). However, because it did not include a HC group, this study could not distinguish hippocampal atrophy due to normal aging from that due to PD degeneration.

The aims of the present study were: (1) to investigate longitudinal changes in hippocampal segments in a sample of PD patients classified according to their baseline cognitive status over a 4-year follow-up; (2) to examine the predictive utility of specific hippocampal subfield volumes as well as total volumes at time 1 to determine changes in memory test scores over time in the PD subgroups; and (3) to investigate the relationship between hippocampal changes over time and memory performance decline.

Based on the previous literature on aging, we would expect that CA1 would be one of the segments atrophied over time, but we would also expect to observe changes in other subfields more specific of PD such as CA2–3. We also hypothesized that the changes in total hippocampal volumes as well as specific segments would explain progressive memory decline.

## Materials and Methods

### Participants

Forty-four PD patients (PD-NC = 28; PD-MCI = 16) from the PD and Movement Disorders Unit, Hospital Clinic (Barcelona, Spain) and 21 HC from the Aging Institute in Barcelona were assessed twice at an interval of 3.8 ± 0.4 years (range: 3.1–5.3).

At time 1, 90 PD patients and 32 HC were recruited between October 2010 and March 2012. Detailed information of the sample can be found in our previous work (Segura et al., 2014). In the present study, only subjects who underwent comprehensive neuropsychological and MRI acquisition at both times were included.

At time 2, two patients underwent deep brain stimulation, five patients and one HC died, 12 PD patients and two controls refused to participate or had moved at follow-up, three PD patients and three controls had developed neurological/psychiatric comorbidities, 15 PD patients had functional impairment and reduced mobility that prevented going to the hospital for MRI scanning, six patients and three HC had MRI motion artifacts or could not finish the scanning protocol and three patients and two HC were excluded due to problems in longitudinal image preprocessing.

Inclusion criteria for patients at time 1 were: (i) fulfilling the UK PD Society Brain Bank diagnostic criteria for PD (Hughes et al., 1992); and (ii) no surgical treatment with deep-brain stimulation. Exclusion criteria for PD patients and HC were: (i) dementia according to the Movement Disorders Society (MDS) criteria (Emre et al., 2007) and to clinical assessment performed by a clinical neurologist (MM, FV, YC); (ii) red flags for atypical parkinsonisms; (iii) Hoehn and Yahr (H&Y) scale (Hoehn and Yahr, 1967) score >3; (iv) young-onset PD; (v) age below 50 years; (vi) presence of severe psychiatric or neurological comorbidity; (vii) low global intellectual quotient estimated by the Vocabulary subtest of the Wechsler Adult Intelligence Scale (scalar score ≤ 7); (viii) Mini Mental State Examination (MMSE) score (Folstein et al., 1975) below 25; (ix) claustrophobia; (x) pathological MRI findings other than mild white matter hyperintensities in the FLAIR sequence; and (xi) MRI artifacts. At time 2, a diagnosis of dementia, H&Y score >3 and MMSE scores below 25 were not considered as exclusion criteria.

Motor symptoms were assessed with the Unified PD Rating Scale motor section (UPDRS-III, Fahn and Elton, 1987). All PD patients were taking antiparkinsonian drugs, consisting of different combinations of L-DOPA, catechol-O-methyltransferase inhibitors, monoamine oxidase inhibitors, dopamine agonists and amantadine. In order to standardize doses, the L-DOPA equivalent daily dose (LEDD) was calculated (Tomlinson et al., 2010).

Written informed consent was obtained from all study participants after full explanation of the procedures. The study was approved by the institutional Ethics Committee from the University of Barcelona (IRB00003099).

### Neuropsychological and Clinical Assessment

The diagnosis of PD-MCI was established in line with MDS task force recommendations (Litvan et al., 2012) as previously described in Segura et al. (2014). The memory domain was assessed with Rey’s Auditory Verbal learning test (RAVLT; Lezak et al., 2012) using total learning (RAVLT total), delayed recall (RAVLT recall) and recognition (RAVLT recognition) scores. Initially, *z-scores* for each test and for each subject were calculated based on the control group’s means and standard deviations (SDs) from time 1. Expected *z-scores* adjusted for age, sex and education for each test and each subject were calculated based on a multiple regression analysis performed in the HC group (Aarsland et al., 2009).

Neuropsychiatric symptoms were evaluated with the Beck Depression Inventory-II (Beck et al., 1996), Starkstein’s Apathy Scale (Starkstein et al., 1992) and Cumming’s Neuropsychiatric Inventory (Cummings et al., 1994).

### Preprocessing of Longitudinal Imaging

MRI data were acquired with a 3T scanner (MAGNETOM Trio, Siemens, Germany) at both times. The scanning protocol included high-resolution 3-dimensional T1-weighted images acquired in the sagittal plane (TR = 2,300 ms, TE = 2.98 ms, TI = 900 ms, 240 slices, FOV = 256 mm; 1 mm isotropic voxel) and an axial FLAIR sequence (TR = 9,000 ms, TE = 96 ms).

Cross sectional preprocessing of both times was estimated using the automated FreeSurfer stream (version 5.1^1^). Detailed description of FreeSurfer procedures is reported in Segura et al. (2014). In addition, to extract reliable volume and thickness estimates, images were automatically processed with FreeSurfer’s longitudinal stream (Reuter et al., 2012). Specifically, an unbiased within-subject template space and image is created using robust, inverse consistent registration (Reuter et al., 2010). Several processing steps, such as skull stripping, Talairach transforms, atlas registration as well as spherical surface maps and parcellations are then initialized with common information from the within-subject template, significantly increasing reliability and statistical power (Reuter et al., 2012).

**Figure 1:**
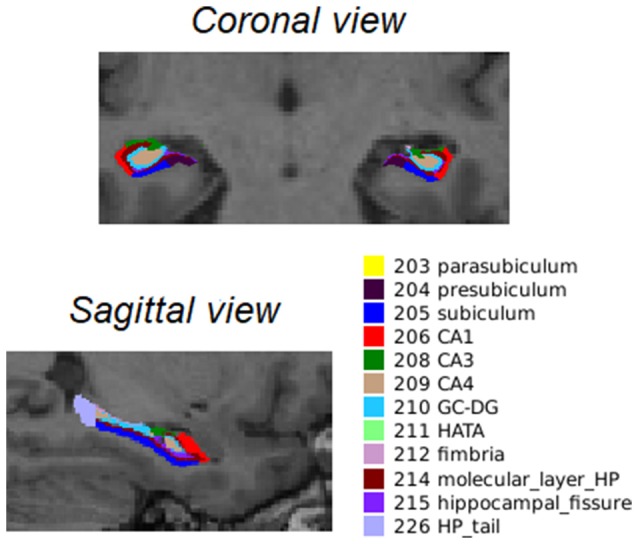
Coronal and sagittal view of the 12 bilateral segments in which the hippocampus was automatically segmented as described by Iglesias et al. (2015). Abbreviations: CA, cornu ammonis; GC-DG, granule cells in the molecular layer of the dentate gyrus; HATA, hippocampal amygdala transition area; HP_tail, hippocampal tail.

After longitudinal preprocessing, FreeSurfer version 6.0 was used to segment the hippocampal subfields^2^. For a visual representation of the hippocampal segments, see Figure 1.

Ratios were calculated for all hippocampal segment volumes to global hippocampal volumes ((lh or rh segments/lh or rh hippocampus)*100). Global hippocampal to estimated total intracranial volume ratios (eTIV, (lh or rh hippocampus/eTIV)*100) were also calculated.

### Statistical Analysis

#### Cross-Sectional Analyses

Group differences in demographic variables and disease outcomes were analyzed with Kruskal-Wallis tests followed by Mann-Whitney-Wilcox’s pairwise comparisons and Bonferroni correction for quantitative measures. Pearson’s chi-squared test was used where appropriate for categorical measures. These analyses were conducted using RStudio Version 1.1.419 (RStudio Team, 2015); information on the libraries and functions can be found in Supplementary Methods S1.

A general linear model and Monte Carlo permutation testing with 10,000 iterations were applied to perform group comparisons of hippocampal volumes ratios at time 1 using Matlab R2017a (The MathWorks Inc., Natick, MA, USA). To control type-I errors, a Bonferroni correction was applied. Age and years of education were included as covariates of no interest.

#### Repeated Measures Analyses

Repeated measures analyses were also conducted with Matlab as described above. Main effects of time and group-by-time interaction were tested on clinical variables such as UPDRS, LEDD and neuropsychiatric symptoms, on memory performance scores and hippocampal subfield volumes between PD groups and HC. Age at time 1 and years of education were used as covariates of no interest in longitudinal hippocampal subfield analyses. For repeated memory score analyses, we used the z-scores adjusted for age, education and sex as described above. Bonferroni correction was applied to all analyses.

#### Multiple Regression Analyses in the PD Patient Sample

Two different multiple linear regression analyses were performed using two models. As a response variable, both models included the difference between time 2 minus time 1 RAVLT raw scores in total learning, recall and recognition. The first model included age at time 1, years of education and hippocampal segments as predictors. The second model included age at time 1, years of education and whole hippocampal volumes as predictors.

First, we assessed the predictive utility of hippocampal ratios at time 1 to explain the variability in memory performance changes. Second, we included the change in hippocampal ratios (time 2 − time 1) as explanatory variables of memory change.

A stepwise model selection by Akaike information criterion (AIC) was applied on the multiple linear regression models described above. This method picks the best-fitted model that most adequately describes an unknown, high dimensional reality (Zhang, 2016). Resulting hippocampal structures that best described prediction of changes in memory performance can be found in Supplementary Methods S2.

Finally, only multiple regression models with statistical significance are reported. Within the RAVLT recall models, an ANOVA was used to test if there were significant differences between the segments model and the global volumes model.

## Results

### Demographic Characteristics and Clinical Evolution

There were no significant differences in scan interval between groups (*H* = 0.013; *P* = 0.994). Thus, involution of the hippocampus and memory can be directly compared. Moreover, the groups had similar disease duration and H&Y staging scores. Although not significant at *p* < 0.05, subjects in the HC group were older than those in the PD subgroups; for this reason, age was included as a covariate in group analyses and as a variable of interest in multiple regression models as described in the “Materials and Methods” section (Table 1).

**Table 1:** Demographic and clinical characteristics of the sample at both times.

	PD-NC *n* = 28	PD-MCI *n* = 16	PD whole sample *n* = 44	Controls *n* = 21	Test stats	*P*-value
**Age, years, median (IQ range)**						
Time 1	59.0 (11.8)	61.5 (17.3)	60.0 (10.5)	67.0 (13.0)	*H* = 6.226^1^ *U* = 593.0^2^	0.044^1^ 0.067^2^
Time 2	63.5 (12.5)	66.0 (15.8)	64.5 (12.0)	70.0 (12.0)	*H* = 5.795^1^ *U* = 590.0^2^	0.055^1^ 0.073^2^
Education, years, median (IQ range)	13.0 (8.3)	10.0 (7.5)	12.0 (8.3)	10.0 (7.0)	*H* = 1.583^1^ *U* = 405.0^2^	0.453^1^ 0.426^2^
Sex, female, *n* (%)	8 (28.6)	6 (37.5)	14 (31.8)	10 (47.6)	X^2^ = 1.872^1^ X^2^ = 0.921^2^	0.392^1^ 0.337^2^
**Disease duration, years, median (IQ range)**						
Time 1	6.0 (5.0)	6.0 (9.5)	6.0 (7.5)	NA	*U* = 197.5	0.759
Time 2	9.0 (4.0)	8.0 (8.3)	9.0 (6.3)	NA	*U* = 248.0	0.565
Age of onset, years, median (IQ range)	53.5 (15.8)	55.3 (15.8)	54.0 (15.8)	NA	*U* = 180.0	0.294
**Hoehn & Yahr stage, *n* 1/1.5/2/2.5/3/4**						
Time 1	11/1/12/2/2/0	6/0/8/1/1/0	17/1/20/3/3/0	NA	X^2^ = 0.718	0.949
Time 2	3/0/14/0/11/0	3/0/5/0/7/1	6/0/19/0/18/1	NA	X^2^ = 3.111	0.375

*IQ range, interquartile range; NA, not applicable; PD-MCI, Parkinson’s disease with mild cognitive impairment; PD-NC, Parkinson’s disease with normal cognition. P-values are from Kruskal-Wallis test followed by Mann-Whitney pairwise test and Bonferroni correction for continuous variables and chi-squared test for categorical variables. ^1^Test stats and P-values of PD subgroups and HC comparisons. ^2^Test stats and P-values of the PD collapsed sample and HC comparisons*.

Table 2 summarizes the time effects observed for the clinical measures. The collapsed PD sample had significant decline over time in global cognition scores, increased motor severity as measured by the UPDRS-III and increased neuropsychiatric symptoms. All the PD groups had increased neuropsychiatric symptoms and PD-NC also showed increased depression scores. No significant changes were seen in apathy scale scores.

**Table 2:** Repeated clinical measures.

	PD-NC *n* = 28	PD-MCI *n* = 16	PD all sample *n* = 44	Controls *n* = 21	Time effects			
					PD-NC	PD-MCI	PD all sample	Controls
**Mini Mental State Examination, mean (SD)**								
Time 1	29.5 (0.7)	28.7 (1.5)	29.2 (1.1)	29.8 (0.4)	*t* = 1.366; *P* = 0.055	*t* = 2.957; *P* = 0.050	*t* = 2.843; *P* = 0.007*	*t* = 1.291; *P* = 0.052
Time 2	29.1 (1.0)	27.6 (3.4)	28.6 (2.3)	29.3 (0.9)
**UPDRS part III, mean (SD)**								
Time 1	13.9 (9.2)	11.8 (11.0)	13.1 (9.8)	NA	*t* = 1.533; *P* = 0.052	NS	*t* = 2.073; *P* = 0.022*	NA
Time 2	17.8 (9.0)	17.4 (12.7)	17.7 (10.3)	NA
**LEDD, mg, mean (SD)**								
Time 1	700.8 (470.6)	675.6 (535.2)	691.6 (489.0)	NA	NS	NS	NS	NA
Time 2	720.5 (388.2)	693.3 (481.1)	710.6 (419.0)	NA
**Beck Depression Inventory II, mean (SD)**								
Time 1	7.3 (4.9)	11.1 (6.3)	8.7 (5.7)	7.0 (5.4)	*t* = 1.508; *P* = 0.043*	NS	NS	NS
Time 2	8.5 (7.4)	10.0 (6.3)	9.1 (7.0)	5.3 (4.7)
**Starkstein’s Apathy Scale, mean (SD)**								
Time 1	10.6 (5.7)	14.3 (8.7)	11.9 (7.0)	9.1 (5.6)	NS	NS	NS	NS
Time 2	11.4 (6.6)	14.1 (9.4)	12.4 (7.7)	9.3 (5.6)
**Cummings’ Neuropsychiatric Inventory, mean (SD)**								
Time 1	4.5 (7.2)	9.1 (11.8)	6.2 (9.3)	1.9 (3.6)	*t* = 1.993; *P* = 0.041*	*t* = 2.343; *P* = 0.041*	*t* = 3.016; *P* = 0.006*	NS
Time 2	7.1 (8.8)	13.1 (11.6)	9.3 (10.2)	2.3 (2.6)				

LEDD, L-DOPA equivalent daily dose; NA, not applicable; PD-MCI, Parkinson’s disease with mild cognitive impairment; PD-NC, Parkinson’s disease with normal cognition; SD, standard deviation; UPDRS part III, Unified Parkinson’s Disease Rating Scale motor section. NS, non-significant. *P-values < 0.05 after Bonferroni correction.

### Longitudinal Changes in Hippocampal Segments

Longitudinally, both PD-NC and PD-MCI as well as the PD collapsed sample showed a significant time effect in the right whole hippocampus. Regarding time effects in hippocampal segments, the right CA1 displayed a significant effect of time in all groups of PD patients and HC. Moreover, the left hippocampal tail (HP_tail) and right parasubiculum had significant decreases in the PD collapsed sample and PD-NC patients. Significant group-by-time interaction was seen in the right parasubiculum in the contrast HC vs. PD-NC and PD-NC vs. PD-MCI. Means and SDs of hippocampal segments can be found in Supplementary Table S1; test stats and uncorrected *P*-values can be found in Table 3. After Bonferroni correction, *P*-values were not significant.

**Table 3:** Time effects and group-by-time interaction for significant hippocampal segments.

	Time effects				Interaction time × group			
	PD-NC	PD-MCI	PD all sample	Controls	HC > PD-NC	HC > PD-MCI	PD-NC > PD-MCI	HC > PD all sample
Left hippocampal tail	*t* = 1.923; *P* = 0.019	NS	*t* = 1.764; *P* = 0.028	NS				
Right CA1	*t* = 1.682; *P* = 0.030	*t* = 2.082; *P* = 0.019	*t* = 1.864 *P* = 0.027	*t* = 2.037 *P* = 0.023				
Right parasubiculum	*t* = 1.985 *P* = 0.035	NS	*t* = 1.932 *P* = 0.026	NS	*t* = 1.498; *P* = 0.031		*t* = −2.574 *P* = 0.020	
Right whole hippocampus	*t* = 1.614; *P* = 0.031	*t* = 1.539; *P* = 0.040	*t* = 1.626; *P* = 0.030	NS				

*CA, Cornu Ammonis; HC, healthy controls; NS, non-significant; PD-MCI, Parkinson’s disease mild cognitive impairment; PD-NC, Parkinson’s disease normal cognition*.

### Predictive Utility of Hippocampal Volumes in Memory Decline

#### Memory Decline

Regarding memory performance, all z-scores were lower at follow-up. The collapsed PD sample showed significant decline in all variables mainly due to progressive impairment in the PD-NC group. PD-NC had a significant decrease in RAVLT total learning and recognition (Table 4). For RAVLT total learning, there was a significant group-by-time interaction between PD-NC and HC (*t* = 2.301; *P* = 0.013; *P*-corrected < 0.05). For RAVLT recognition, the interaction was significant for PD-NC and HC (*t* = 2.969; *P* < 0.001; *P*-corrected < 0.05) and for all PD sample vs. controls (*t* = 2.713; *P* < 0.001; *P*-corrected = 0.05).

**Table 4:** Repeated measures analysis of memory performance.

	PD-NC *n* = 28 mean (SD)	PD-MCI *n* = 16 mean (SD)	PD all sample *n* = 44 mean (SD)	Controls *n* = 21 mean (SD)	Time effects			
					PD-NC	PD-MCI	PD all sample	Controls
**RAVLT total learning**								
Time 1	0.14 (1.12)	−1.52 (0.80)	−0.47 (1.29)	0.00 (0.81)	*t* = 3.836; *P* = 0.001*	NS	*t* = 3.315; *P* = 0.001*	NS
Time 2	−0.58 (1.30)	−1.66 (1.25)	−0.97 (1.37)	−0.06 (0.73)			
**RAVLT delayed recall**								
Time 1	−0.15 (1.12)	−1.30 (0.94)	−0.57 (1.19)	0.06 (0.90)	NS	NS	*t* = 1.956; *P* = 0.034	NS
Time 2	−0.46 (1.38)	−1.70 (1.10)	−0.91 (1.41)	−0.04 (0.74)			
**RAVLT recognition**								
Time 1	0.20 (1.04)	−0.95 (2.23)	−0.22 (1.65)	−0.17 (0.76)	*t* = 4.059; *P* < 0.001*	NS	*t* = 4.178; *P* < 0.001*	NS
Time 2	−1.38 (2.85)	−1.76 (1.86)	−1.51 (2.52)	0.01 (0.94)			

NS, not significant; PD-MCI, Parkinson’s disease with mild cognitive impairment; PD-NC, Parkinson’s disease with normal cognition; RAVLT, Rey’s auditory verbal learning test. Means are adjusted z-scores by age, years of education and sex. *Indicates statistical significance (P-values < 0.05) after Bonferroni correction.

#### Hippocampal Volume Ratios at Time 1 as Predictors of Memory Change Over Time

The first multiple regression approach investigated whether hippocampal volume ratios at time 1 can be good predictors of memory performance change.

For RAVLT total changes over time, using whole hippocampal volume ratios as predictors, the right whole hippocampus was a significant predictive variable (*R*^2^ = 0.16; adjusted *R*^2^ = 0.14; *F* = 8.074; *P* = 0.007). However, the segments model for change over time was not significant (*R*^2^ = 0.26; adjusted *R*^2^ = 0.07; *F* = 1.332; *P* = 0.257).

For RAVLT recall change, the significant predictive variables were age, left CA3, right CA4, left parasubiculum, right subiculum, right fimbria, right HP_tail, right fissure and left molecular layer (*R*^2^ = 0.69; adjusted *R*^2^ = 0.56; *F* = 5.634; *P* < 0.001). Considering that the bilateral whole hippocampus was a significant predictor (*R*^2^ = 0.23; adjusted *R*^2^ = 0.19; *F* = 5.960; *P* = 0.005), there were significant differences between the segments and the global volumes models (*F* = 4.539; *P* = 0.001).

Regarding RAVLT recognition change, the bilateral CA1, right CA4, bilateral subiculum and right Hippocampal Amygdala Transition Area (HATA) were significant predictive variables (*R*^2^ = 0.44; adjusted *R*^2^ = 0.27; *F* = 2.557; *P* = 0.021).

Detailed information of the multiple regression models for each test can be found in Supplementary Table S2.

#### Relationship Between Hippocampal Volume Ratio Change and Memory Decline

The second multiple regression approach aimed to investigate whether changes in hippocampal volume ratios can explain changes in memory performance.

Changes in right fimbria, right HP_tail and left fissure were significant explanatory variables of changes in RAVLT recall scores (*R*^2^ = 0.67; adjusted *R*^2^ = 0.45; *F* = 3.093; *P* = 0.005). In the global model, the left hippocampus was the only significant variable (*R*^2^ = 0.20; adjusted *R*^2^ = 0.14; *F* = 3.240; *P* = 0.032). There were significant differences between the two models (*F* = 2.659; *P* = 0.015).

Finally, for RAVLT recognition, when considering the whole structure (*R*^2^ = 0.14; adjusted *R*^2^ = 0.12; *F* = 6.589; *P* = 0.014), the left hippocampus was a significant predictor.

Information regarding the multiple regression models for each test can be found in Supplementary Table S3.

## Discussion

The main findings of the present study were: (1) the right CA1 was sensitive to time effects in normal aging and in PD with NC and with MCI; (2) volume decrements in right whole hippocampus volume as well as specific regional volumes were only found in PD; and (3) hippocampal subfields were better predictors of delayed verbal memory recall decline than global hippocampal volumes.

The right CA1 showed a significant time effect for all PD groups and for HC. No significant group-by-time interactions were found. Therefore, the changes observed seem to be due to aging effects rather than specific of PD. CA1 has been reported as one of the regions with the earliest and strongest involvement over time in AD (Small et al., 2011), being useful to discriminate healthy subjects from those with MCI (Mueller et al., 2010) and to predict conversion from MCI to AD (Apostolova et al., 2010). Indeed, early neuropathological studies have described a high susceptibility to the accumulation of amyloid-β in CA1 both in a mouse model and humans (Furcila et al., 2018).

Cross-sectional studies have reported that the head of the hippocampus is the most vulnerable region in normal aging (Ta et al., 2012), in non-demented PD (Ibarretxe-Bilbao et al., 2008) and in demented PD patients (Bouchard et al., 2008; Ibarretxe-Bilbao et al., 2008) but demented patients also revealed posterior hippocampal atrophy (Ibarretxe-Bilbao et al., 2008). In the present longitudinal study, in addition to the decrements described above, we also found significant volume decrements in the left HP_tail for the PD-NC group, suggesting that specific posterior hippocampal atrophy takes place at earlier stages of the disease.

When considering global hippocampal volumes, the right hippocampus had specific time effects in all PD subgroups. This pattern is different from what occurs in amnestic MCI and AD. In a meta-analysis of 14 studies it has been reported that although in both MCI and AD there are progressive bilateral reductions, the effect size is greater for the left hemisphere when compared with the right (Shi et al., 2009). A recent work by Yue et al. (2018) reported hippocampal asymmetry in MCI patients and individuals with subjective cognitive decline compared with HC where the left hemisphere was more atrophic than the right.

The right parasubiculum was also sensitive to time effects in the PD-NC group. Volume decrements in this region were significantly higher than those observed in the HC and the PD-MCI group as demonstrated by the significant group-by-time interaction. The parasubiculum is a small hippocampal structure that is usually studied together with the subiculum and parasubiculum. Therefore, there is not much previous literature using MRI techniques describing the implication of this structure in aging or neurodegenerative processes. However, we could speculate that the volume decrements found in the right parasubiculum might be related to parietal atrophy through transneuronal degeneration. The parasubiculum has direct projections to medial parieto-temporal regions involved in visuospatial processes (Dalton and Maguire, 2017). In PD, there is a structural temporo-parietal atrophy suggested as a marker of cognitive decline (Segura et al., 2014). More specifically, medial parietal atrophy has been linked to visuospatial impairment in PD patients (Garcia-Diaz et al., 2014).

Regarding memory performance, hippocampal segment volumes as well as whole hippocampal volumes at baseline have been reported as significant predictors of verbal memory delayed recall changes (Beyer et al., 2012). In our study, the segments model explained 56% of the variability, whereas the whole hippocampal volumes model only explained 19%. In line with this, the right fimbria, right HP_tail and left fissure volume changes over time were linked to changes in RAVLT recall changes in a model that explained almost 50% of the variance. By contrast, the whole hippocampal model was more useful to predict changes in RAVLT total learning scores, although these models did not explain much variability. We could speculate that declines in verbal memory learning would be related to hippocampal-neocortical connectivity more than based on structural changes in the hippocampal formation *per se* (Fjell et al., 2016). Finally, models including RAVLT recognition changes also explained less than 50% of the variance, with the segments model explaining more variance than the global volumes model.

The strengths of the present study are: the inclusion of a control group allowed us to compare hippocampal atrophy in PD with atrophy that occurs in elderly healthy subjects as part of the aging process. Also, the use of novel neuroimaging automated pipelines to accurately segment the hippocampus to investigate regional vulnerability. FreeSurfer’s pipeline is based on *ex vivo* 7T images to manually segment the hippocampus in order to create the statistical atlas; and it has been recently proved to have a good test-retest reliability over time (Worker et al., 2018). However, the authors (Iglesias et al., 2015) recommend caution on the interpretation of results involving the internal subfields such as the CA4, molecular layer or the Granule cells in the molecular layer of the DG. To the best of our knowledge, there is no study that compares the subfields overlap with other manual or automated segmentations methods.

The most important limitation would be the small sample size due to a high attrition rate in the Parkinson’s cohort. To overcome this problem, which affects generalization of the results, larger multicentric studies in PD should help clarify the progressive pattern of degeneration in the hippocampus. There are few longitudinal studies in PD cohorts performing MRI assessments over more than 1.5 years of follow-up. The work of Ulla et al. (2013) followed a cohort of PD patients over 3 years and they also reported an attrition rate of 50%. Multicentric longitudinal initiatives are particularly common in AD, such as the ADNI database^3^ or the AIBL initiative. In the longitudinal AIBL cohort, almost 60% of the participants returned to MRI and PET follow-up scans (Doré et al., 2013). This relatively higher percentage could be explained because elder controls and AD patients have less motor impairment than PD patients. Moreover, we would like to highlight as a frequent limitation of longitudinal studies that participants with worse disease prognosis, with more depressive/apathetic symptoms or with greater functional impairment in their daily living are more likely to be lost to follow-up.

It could be also mentioned that, due to the exploratory nature of the hippocampal subfield study, we report uncorrected *P*-values. These results should thus be interpreted with caution. This limitation is common to all studies using these new hippocampal segmentations; however, exploratory analyses are necessary to progress in neuroimaging research.

In conclusion, besides regional vulnerability in hippocampus degeneration dependent in part of aging, we found specific hippocampal regions that were more sensitive to time effects in PD. The right global hippocampus also seems to be more vulnerable than the left. Finally, specific hippocampal segment volumes were found to be good markers of verbal delayed recall performance decline over time.

## Author Contributions

CJ contributed in the design of the study. CU, AA and AC contributed to the analysis of the data and CU, BS, HB, AA, AC, MM, FV, YC and CJ contributed to the interpretation of the data. CU and CJ contributed to the draft of the article. CU, BS, HB, AA, AC, MM, FV, YC, NB and CJ revised the manuscript critically for important intellectual content and approved the final version of the manuscript.

## Conflict of Interest Statement

The authors declare that the research was conducted in the absence of any commercial or financial relationships that could be construed as a potential conflict of interest.

## Footnotes

^1^https://surfer.nmr.mgh.harvard.edu

^2^https://surfer.nmr.mgh.harvard.edu/fswiki/LongitudinalHippocampalSubfields

^3^http://adni.loni.usc.edu/
