# Supplementary material for: Differential Progression of Regional Hippocampal Atrophy in Aging and Parkinson’s Disease
Source: Front Aging Neurosci. 2018 Oct 11;10:325. doi: 10.3389/fnagi.2018.00325 (PMC6193198; doi:10.3389/fnagi.2018.00325)
Supplement: Supplementary file 1 [file Table_1.docx]

Supplementary Material

Differential progression of regional hippocampal atrophy in aging and Parkinson’s disease

Carme Uribe, MSc^1^; Barbara Segura, PhD^1^; Hugo Cesar Baggio, MD, PhD^1^; Anna Campabadal, MSc^1^; Anna Isabel Garcia-Diaz, PhD^1^; Alexandra Abos, MSc^1^; Yaroslau Compta, MD, PhD^2,3,4^; Maria Jose Marti, MD, PhD^2,3,4^; Francesc Valldeoriola, MD, PhD^2,3,4^; Nuria Bargallo, MD, PhD^5^; Carme Junque, PhD^1,2,3^*.

^1^Medical Psychology Unit, Department of Medicine. Institute of Neuroscience, University of Barcelona. Barcelona, Catalonia, Spain.

^2^Institute of Biomedical Research August Pi i Sunyer (IDIBAPS). Barcelona, Catalonia, Spain.

^3^Centro de Investigación Biomédica en Red sobre Enfermedades Neurodegenerativas (CIBERNED), Hospital Clínic de Barcelona. Barcelona, Spain.

^4^Parkinson’s Disease and Movement Disorders Unit, Neurology Service, Hospital Clínic de Barcelona. Institute of Neuroscience, University of Barcelona, Barcelona, Catalonia, Spain.

^5^Centre de Diagnòstic per la Imatge, Hospital Clínic, Barcelona, Catalonia, Spain.

*** Correspondence:**Prof. Carme Junque

Medical Psychology Unit, Department of Medicine. University of Barcelona

Casanova 143 (08036) Barcelona, Spain

Phone: (+34) 93 402 45 70 // Fax: (+34) 93 403 52 94 // E-mail: [cjunque@ub.edu](mailto:cjunque@ub.edu)

# Supplementary Methods 1

Libraries and functions used for the statistical analysis using R:

| *Demographic and clinical variables.*  *Descriptives* | **psych** library: describe.by() function for means, medians and SD.  **stats** library: IQR() function for interquartile range. |
| --- | --- |
| *Demographical and clinical variables.*  *Test stats* | **stats** library:  chisq.test() function for chi-squared test;  kruskal.test() function  pairwise.wilcox.test() for multiple comparisons with Bonferroni P-adjusted. |
| *Multiple linear regression model* | **stats** library: lm() function and anova() to test for differences between segments and total volumes models.  **MASS** library: stepAIC() function to fit the best model. |

# Supplementary Methods 2

Multiple linear regression models resulting from stepwise Akaike information criteria:

|  | **time 1 hippocampal volumes ratios** | **time 2 minus time 1 hippocampal volumes ratios** |
| --- | --- | --- |
| *Model 1 hippocampal segments* | | |
| **RAVLT total learning** | lh CA3 + rh CA4 + bl CA1 + rh subiculum + rh presubiculum + bl HATA + rh hippocampal tail | education + bl CA1 + bl CA3 + rh CA4 + bl subiculum + bl presubiculum + bl parasubiculum + bl HATA + bl fimbria + bl hippocampal tail + bl fissure + bl molecular layer + bl GC-ML-DG |
| **RAVLT recall** | age + education + lh CA3 + rh CA1 + rh CA4 + bl parasubiculum + rh subiculum + rh fimbria + rh hippocampal tail + rh fissure + lh molecular layer | education + lh CA1 + lh CA3 + bl CA4 + lh subiculum + bl presubiculum + lh parasubiculum + lh HATA + bl fimbria + bl hippocampal tail + lh fissure + lh molecular layer + lh GC-ML-DG |
| **RAVLT recognition** | bl CA1 + rh CA4 + bl subiculum + rh HATA + bl hippocampal tail + bl molecular layer | age + education + bl CA1 + bl CA3 + bl CA4 + bl subiculum + bl presubiculum + bl parasubiculum + bl HATA + bl fimbria + bl hippocampal tail + bl fissure + bl molecular layer |
| *Model 2 whole hippocampi* | | |
| **RAVLT total learning** | rh whole hippocampus | age + lh whole hippocampus |
| **RAVLT recall** | bl whole hippocampus | age + bl whole hippocampus |
| **RAVLT recognition** | No prediction model | lh whole hippocampus |

# Supplementary Table 1 Means and SD of hippocampal segments

|  | | **PD-NC**  **n = 28**  **mean (SD)** | **PD-MCI**  **n = 16**  **mean (SD)** | **PD all sample**  **n = 44**  **mean (SD)** | **Controls**  **n = 21**  **mean (SD)** |
| --- | --- | --- | --- | --- | --- |
| *Left hippocampal tail* | **time 1** | 15.0 (1.5) | 13.8 (1.4) | 14.6 (1.6) | 14.6 (1.6) |
|  | **time 2** | 14.8 (1.6) | 13.9 (1.5) | 14.5 (1.6) | 14.6 (1.9) |
| *Right hippocampal tail* | **time 1** | 15.3 (1.3) | 15.0 (1.6) | 15.2 (1.4) | 15.5 (1.7) |
|  | **time 2** | 15.1 (1.1) | 15.0 (1.7) | 15.1 (1.3) | 15.3 (1.8) |
| *Left CA1* | **time 1** | 17.9 (0.9) | 18.7 (0.9) | 18.2 (1.0) | 18.3 (1.0) |
|  | **time 2** | 18.3 (1.0) | 18.8 (0.9) | 18.5 (1.0) | 18.3 (1.0) |
| *Right CA1* | **time 1** | 18.2 (1.1) | 18.6 (0.7) | 18.4 (1.0) | 18.1 (0.9) |
|  | **time 2** | 18.5 (1.0) | 18.7 (1.0) | 18.6 (0.9) | 18.2 (1.1) |
| *Left CA3* | **time 1** | 6.3 (0.6) | 6.4 (0.6) | 6.3 (0.6) | 6.7 (0.7) |
|  | **time 2** | 6.4 (0.6) | 6.4 (0.7) | 6.4 (0.6) | 6.7 (0.7) |
| *Right CA3* | **time 1** | 6.7 (0.7) | 6.8 (0.5) | 6.7 (0.6) | 6.8 (0.7) |
|  | **time 2** | 6.8 (0.6) | 6.7 (0.5) | 6.8 (0.6) | 6.9 (0.8) |
| *Left CA4* | **time 1** | 8.4 (0.5) | 8.1 (0.5) | 8.3 (0.5) | 8.4 (0.5) |
|  | **time 2** | 8.4 (0.5) | 8.2 (0.6) | 8.3 (0.6) | 8.4 (0.5) |
| *Right CA4* | **time 1** | 8.6 (0.5) | 8.3 (0.5) | 8.5 (0.5) | 8.6 (0.5) |
|  | **time 2** | 8.7 (0.5) | 8.4 (0.4) | 8.6 (0.5) | 8.6 (0.5) |
| *Left subiculum* | **time 1** | 11.9 (0.9) | 11.9 (0.7) | 11.9 (0.8) | 11.6 (0.7) |
|  | **time 2** | 11.8 (0.9) | 12.0 (0.8) | 11.9 (0.9) | 11.6 (0.8) |
| *Right subiculum* | **time 1** | 11.7 (0.8) | 11.7 (0.7) | 11.7 (0.8) | 11.5 (0.7) |
|  | **time 2** | 11.7 (0.9) | 11.8 (0.8) | 11.7 (0.8) | 11.5 (0.7) |
| *Left presubiculum* | **time 1** | 8.5 (1.1) | 8.9 (0.9) | 8.6 (1.0) | 8.4 (0.9) |
|  | **time 2** | 8.4 (1.1) | 8.8 (0.8) | 8.5 (1.1) | 8.4 (0.8) |
| *Right presubiculum* | **time 1** | 7.8 (1.0) | 7.9 (0.7) | 7.8 (0.9) | 7.8 (0.7) |
|  | **time 2** | 7.7 (0.9) | 7.8 (0.8) | 7.7 (0.9) | 7.7 (0.8) |
| *Left parasubiculum* | **time 1** | 1.7 (0.4) | 2.0 (0.5) | 1.8 (0.4) | 1.7 (0.5) |
|  | **time 2** | 1.7 (0.4) | 2.1 (0.6) | 1.9 (0.5) | 1.7 (0.5) |
| *Right parasubiculum* | **time 1** | 1.6 (0.3) | 1.6 (0.4) | 1.6 (0.3) | 1.6 (0.5) |
|  | **time 2** | 1.6 (0.3) | 1.7 (0.4) | 1.6 (0.4) | 1.6 (0.5) |
| *Left fimbria* | **time 1** | 2.3 (0.6) | 2.3 (0.5) | 2.3 (0.6) | 2.1 (0.4) |
|  | **time 2** | 2.1 (0.6) | 2.2 (0.6) | 2.2 (0.6) | 2.1 (0.3) |
| *Right fimbria* | **time 1** | 1.8 (0.5) | 2.0 (0.5) | 1.8 (0.5) | 1.8 (0.5) |
|  | **time 2** | 1.7 (0.5) | 1.8 (0.6) | 1.7 (0.5) | 1.8 (0.5) |
| *Left fissure* | **time 1** | 4.5 (0.9) | 4.9 (1.0) | 4.6 (0.9) | 4.8 (0.9) |
|  | **time 2** | 4.7 (1.0) | 5.3 (1.2) | 4.9 (1.1) | 5.1 (0.8) |
| *Right fissure* | **time 1** | 4.3 (0.9) | 5.1 (1.2) | 4.6 (1.1) | 4.8 (0.9) |
|  | **time 2** | 4.9 (1.0) | 5.5 (1.1) | 5.1 (1.1) | 5.1 (0.9) |
| *Left HATA* | **time 1** | 1.6 (0.2) | 1.6 (0.2) | 1.6 (0.2) | 1.7 (0.2) |
|  | **time 2** | 1.6 (0.2) | 1.6 (0.3) | 1.6 (0.3) | 1.7 (0.2) |
| *Right HATA* | **time 1** | 1.6 (0.2) | 1.6 (0.2) | 1.6 (1.2) | 1.6 (0.2) |
|  | **time 2** | 1.6 (0.2) | 1.6 (0.3) | 1.6 (0.2) | 1.6 (0.3) |
| *Left molecular layer* | **time 1** | 17.2 (0.4) | 17.3 (0.5) | 17.2 (0.5) | 17.3 (0.4) |
|  | **time 2** | 17.2 (0.4) | 17.1 (0.5) | 17.2 (0.5) | 17.2 (0.4) |
| *Right molecular layer* | **time 1** | 17.3 (0.5) | 17.4 (0.6) | 17.3 (0.6) | 17.3 (0.5) |
|  | **time 2** | 17.3 (0.5) | 17.3 (0.5) | 17.3 (0.5) | 17.2 (0.5) |
| *Left GC-ML-DG* | **time 1** | 9.2 (0.5) | 9.0 (0.4) | 9.1 (0.5) | 9.3 (0.5) |
|  | **time 2** | 9.2 (0.5) | 9.0 (0.5) | 9.1 (0.5) | 9.3 (0.5) |
| *Right GC-ML-DG* | **time 1** | 9.5 (0.5) | 9.2 (0.5) | 9.3 (0.5) | 9.4 (0.5) |
|  | **time 2** | 9.5 (0.5) | 9.2 (0.5) | 9.4 (0.5) | 9.4 (0.6) |
| *Left whole hippocampus* | **time 1** | 0.3 (0.0) | 0.2 (0.0) | 0.2 (0.0) | 0.2 (0.0) |
|  | **time 2** | 0.2 (0.0) | 0.2 (0.0) | 0.2 (0.0) | 0.2 (0.0) |
| *Right whole hippocampus* | **time 1** | 0.3 (0.0) | 0.2 (0.0) | 0.3 (0.0) | 0.3 (0.0) |
|  | **time 2** | 0.3 (0.0) | 0.2 (0.0) | 0.2 (0.0) | 0.2 (0.0) |

CA, Cornu Ammonis; GC-ML-DG, Granule cells in the molecular layer of the dentate gyrus; HATA, Hippocampal Amygdala Transition Area; PD-MCI, Parkinson’s disease mild cognitive impairment; PD-NC, Parkinson’s disease normal cognition; SD, standard deviation.

Values are volumes in mm^3^. Hippocampal segments are ratios to whole hippocampal volumes ((lh or rh segment / lh or rh whole hippocampus) * 100). Whole hippocampus volumes are ratios to estimated total intracranial volume ((lh or rh whole hippocampus / eTIV) * 100).

# Supplementary Table 2 Multiple regression results of time 1 hippocampal volumes ratios as predictors of memory performance change over time

| *Model 1 hippocampal segments* | | | | *Model 2 whole hippocampi* | | |
| --- | --- | --- | --- | --- | --- | --- |
|  | **variables** | | **t-stat (P-value)** | **variables** | | **t-stat (P-value)** |
| RAVLT total learning | Non-significant model | | | right hippocampus | **2.841 (0.007)** | |
| RAVLT recall | age | **-2.310 (0.028)** | | left hippocampus | **-2.610 (0.013)** | |
|  | education | -1.707 (0.098) | | right hippocampus | **3.346 (0.002)** | |
|  | left CA3 | **-3.040 (0.005)** | |  | | |
|  | right CA1 | -1.428 (0.163) | |  |  |  |
|  | right CA4 | **-3.561 (0.001)** | |  |  |  |
|  | left parasubiculum | **-5.825 (<0.001)** | |  |  |  |
|  | right subiculum | **-3.147 (0.004)** | |  |  |  |
|  | right parasubiculum | -0.640 (0.527) | |  |  |  |
|  | right fimbria | **-4.831 (<0.001)** | |  |  |  |
|  | right hippocampal tail | **-5.798 (0.001)** | |  |  |  |
|  | right fissure | **3.195 (0.003)** | |  |  |  |
|  | left molecular layer | **-3.491 (0.001)** | |  |  |  |
| RAVLT recognition | Non-significant model | | | No prediction model | | |

CA, Cornu Ammonis; RAVLT, Rey’s Auditory Verbal Learning test.

Regions that reached significance P<0.05 are marked in bold.

# Supplementary Table 3 Multiple regression results of time 2 minus time 1 hippocampal volumes ratios as explanatory variables of memory changes

| *Model 1 hippocampal segments* | | | | *Model 2 whole hippocampi* | | |
| --- | --- | --- | --- | --- | --- | --- |
|  | **variables** | | **t-stat (P-value)** | **variables** | **t-stat (P-value)** | |
| **RAVLT total learning** | | Non-significant model |  | age | | -1.600 (0.117) |
|  |  |  |  | left hippocampus | | 1.688 (0.099) |
| **RAVLT recall** | | education | 1.299 (0.205) | age | | -1.376 (0.176) |
|  |  | left CA1 | 1.211 (0.237) | left hippocampus | | **2.151 (0.038)** |
|  |  | left CA3 | 1.211 (0.237) | right hippocampus | | -1.556 (0.128) |
|  |  | left CA4 | 1.211 (0.237) |  | | |
|  |  | right CA4 | 1.696 (0.102) |  |  |  |
|  |  | left subiculum | 1.211 (0.237) |  |  |  |
|  |  | left presubiculum | 1.211 (0.237) |  |  |  |
|  |  | left parasubiculum | 1.211 (0.237) |  |  |  |
|  |  | right presubiculum | 1.123 (0.272) |  |  |  |
|  |  | left HATA | 1.211 (0.237) |  |  |  |
|  |  | left fimbria | 1.211 (0.237) |  |  |  |
|  |  | right fimbria | **2.650 (0.014)** |  |  |  |
|  |  | left hippocampal tail | 1.211 (0.237) |  |  |  |
|  |  | right hippocampal tail | **2.986 (0.006)** |  |  |  |
|  |  | left fissure | **-2.888 (0.008)** |  |  |  |
|  |  | left molecular layer | 1.211 (0.237) |  |  |  |
|  |  | left GC-ML-DG | 1.211 (0.237) |  |  |  |
| **RAVLT recognition** | | Non-significant model | | left hippocampus | | **2.567 (0.014)** |

CA, Cornu Ammonis; GC-ML-DG, Granule cells in the molecular layer of the dentate gyrus; HATA, Hippocampal Amygdala Transition Area; RAVLT, Rey’s Auditory Verbal Learning test.

Regions that reached significance P<0.05 are marked in bold.
